# Lamination of Separators to Electrodes using Electrospinning

**DOI:** 10.1371/journal.pone.0227903

**Published:** 2020-01-28

**Authors:** Bernhard Christian Springer, Martin Frankenberger, Karl-Heinz Pettinger

**Affiliations:** Technology Centre for Energy, University of Applied Sciences Landshut, Ruhstorf a. d. Rott, Bavaria, Germany; East China Normal University, CHINA

## Abstract

This study shows the feasibility of the Electrospinning method as a process step for advanced and fast production Li ion cells. Lamination is a key technology for Lithium-ion battery production. It bares different advantages, such as a fast production line speed by fixing the separator to the electrodes. Unfortunately, this technology is inapplicable for separator and electrode formulations not based on thermoplastic binders. Using Electrospinning, this disadvantage can be overcome. In our study, beaded PVDF polymer nanofibres were spun onto a fibre-reinforced, inorganic-filled separator. This modified separator was then laminated onto a NMC111-cathode using a temperature profile of 110/110/120°C within the laminator. After Lamination, the separator was pulled-off again and placed in a SEM to see the adhesive behaviour of the applied polymer. The information gathered with SEM clearly shows a successful lamination of the separator to the electrode.

## Introduction

With the rise of portable consumer electronics over the last decades, lithium-ion battery technology has become one of the most used storage technologies. Beginning in the 1970s, after the commercialization by Sanyo [1] lithium-ion batteries have gained a lot of attention by the scientific community. Despite the progress already made, the technology still has challenges to be solved. Especially for automotive and portable electronics, the fast charging capability of the cells is of interest. Therefore, research on the electrode [2–4], electrolyte [5] and separator [6] materials as well as their corresponding manufacturing steps is conducted to tackle this challenge. Also the manufacturing process of the cells and cell stacks has an impact on the energy density. Three different manufacturing processes for prismatic cells are well known: winding, z-folding and stacking [7]. In case of stacking, lamination can be used to improve the production process speed and precision [7]. Lamination has been known for its simplification of especially the stacking process [8–9]. In addition, Frankenberger et al. showed an improvement of the fast charging capability of laminated single cells [10]. Unfortunately, Lamination cannot be applied to all separators, especially if they contain binders or materials which are not thermoplastic. This leads to an exclusion of a lot of interesting separators from Lamination. To overcome this restraint, the method of Electrospinning of thermoplastic binders is used in this study. The same approach was used by Hun Lee in his PhD thesis, where PVDF polymers were electrospun onto several polyolefin separators [11]. Electrospinning offers several advantages for enabling Lamination: it is easy to use, it can be applied to a wide range of materials, it can tune the morphology of fibres and it can tune the area loading. Especially the last point was important since it is therefore possible to optimize the adhesion and at the same time control a possible blocking of the electrode-separator interface. The aim is therefore to have enough thermoplastic to create adhesion but at the same time keep as much separator surface and pores open and unblocked. Electrospinning produces fibres in the nano- to micrometer range, which can also be modulated in their morphology [12]. Electrospinning is used in many different fields such as Catalysis, Filtration, Sensors, Textiles and Tissue engineering [13].

## Materials and methods

NMC (93 wt%; NM-3102 h, BASF, Germany–former TODA America, USA) is mixed in a planetary mixer (TX-2, INOUE, Japan) with Super C65 carbon (3 wt%; IMERYS, Switzerland–former TIMCAL, Switzerland), KS6L graphite (1 wt%; IMERYS) and PVDF binder (3 wt%; Solef^®^ 5130, SOLVAY, Italy) using N-methyl-pyrrolidone (NMP; Overlack, Germany) as a solvent. The resulting slurry is then single-side coated using a doctor-blade coater in a roll-to-roll process. The electrodes are dried during the coating process in a two-step in-line drying tunnel at a temperature range of 135°C to 150°C. The fibre-reinforced, inorganic-filled separators have been place on to the rotating drum collector of the Inovenso NE300-XP Electrospinning set-up. The set-up used a common rail three-nozzle emitter and a spinning distance of 12 cm at a voltage of 25kV. To ensure a homogeneous coating of fibres, the collector stage was moved continuously parallel to the drum collector axis 60 mm to each side, with a speed of 5 mm/s. The separator was placed on a drum collector, which rotated with 100 rpm during the spinning process. The humidity inside the spinning chamber was controlled to be 30% relative at 25°C. The spinning solution held a polymer concentration of 10 wt % THV 221 (3M) solved in tetrahydrofuran (THF) using a magnetic stirring plate. The solution is stirred at 75°C for 1 h under reflux and afterwards cooled down to room temperature over a period of 1.5 h. The spinning process itself is conducted for only 3 min for each separator sheet. The electrodes are punched into sheets with an area of 5 x 8 cm^2^. The modified separators are cut to rectangular shape with an area of 5.5 x 8.5 cm^2^. Afterwards, the separators are placed with the electrospun side onto the active material of the electrode and inserted in a roller lamination press (BLE 282 D, *ARCOTRONICS Italia S*.*p*.*A*., Italy; Now *MANZ Italy*). Our modified lamination technique is tested for NMC-cathodes, using a roll speed of 1.38 m/min, a line force of 157 N/cm in a temperature profile of 110/110/120°C in the pre-heating unit of this. The thus prepared cathode-separator pair is then ripped apart to investigate the interfaces after lamination. To control the results and to have further information on the distribution of the polymer on the separator, samples are prepared by electrospinning and lamination without a cathode. To further investigate the lamination, a peeling force test using a manual peeling test stand (TPE, *Sauter*) is administered. The measurable force with this set-up is between 0 and 5 N with a resolution of 0.001 N. The samples are therefore taped to the machine using double-sided tape. The electrode is pulled of directly with a self-adhesive tape from the separator. The samples are prepared for SEM by punching out disks of 12 mm diameter. These disks are attached to sample holders using graphite tape. The samples are not sputtered, therefore only a low acceleration voltage is used for taking the pictures.

## Results and discussion

**Fig 1:**
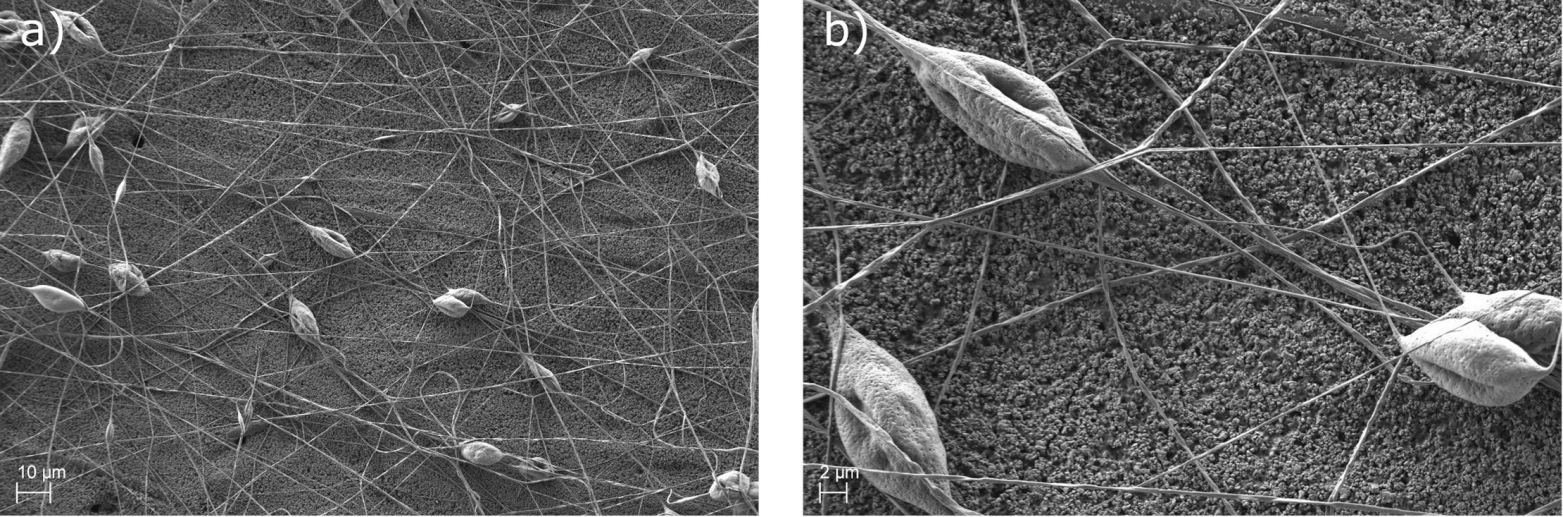
Polymer fibre coating on separator surface. a) Polymer fibre coating after 3 min of electrospinning. The electrospun polymer fibres can be seen very clearly (500-fold magnification). The fibres are loaded from time to time by polymer beads, which are due to the relatively low polymer concentration of the used spinning solution [12, 14]. b) The electrospun coating with 2000-fold magnification.

SEM pictures of the electrospun polymer fibres on the separator for two different magnifications are shown (Fig 1). The pictures show the fibrous, beaded structure of the polymer coating. According to literature [12, 14] these beads occur due to a low concentration of the polymer in the spinning solution. Fig 1A shows the distribution of the fibres and beads on the separator surface. The fibres are spread quite homogeneously, while the beads seem to aggregate. For lamination especially the beads and the distances between are of interest.

These distances seem to be acceptable for the intended lamination application. Fig 1B) shows an example of the beads and fibres found on the separator. It can be seen, that the fibres are relatively homogeneous in thickness; some fibres however are fused during the electrospinning process. The beads show a very rough and porous surface. This is assumed to be an effect of the used solvent THF, which is highly volatile under atmospheric pressure [15]. A second explanation for the porous surfaces of the beads is the relative humidity during the spinning process [16]. To our knowledge, the pores have no effect on the lamination itself.

**Fig 2:**
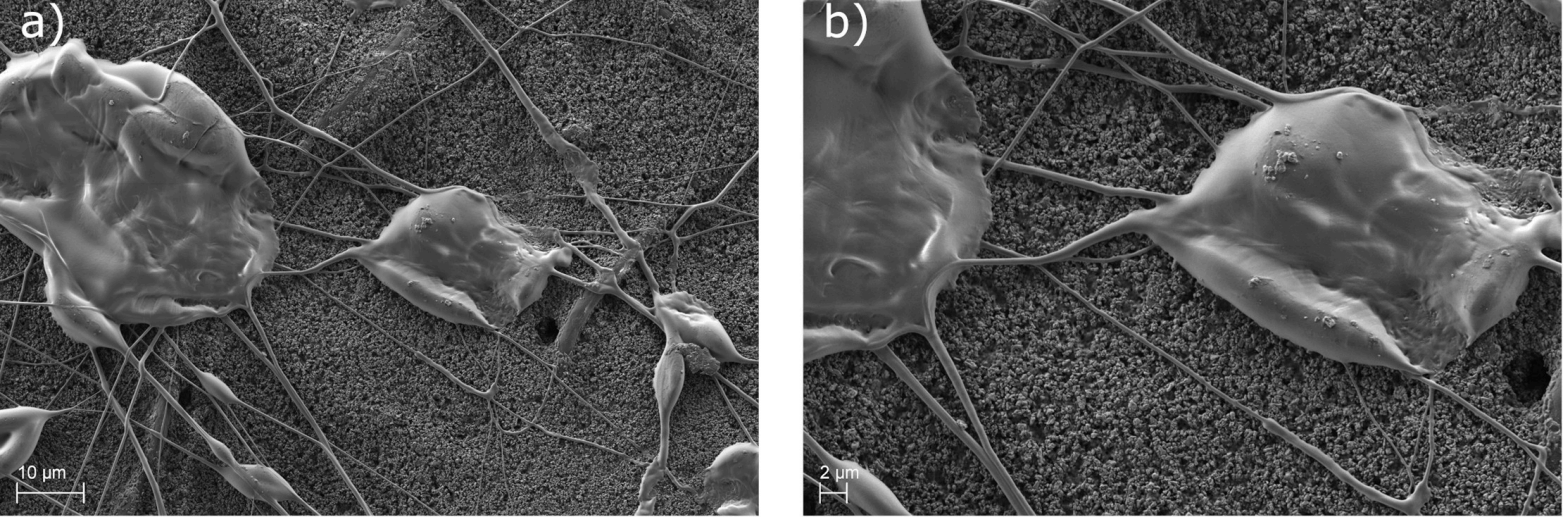
Polymer fibre coating after lamination a) 1000-fold and b) 2000-fold magnification. The lamination is performed as described above. The flattened and molten beads can be seen very clearly.

Fig 2 shows SEM pictures of the separator coated with polymer fibres and laminated according to the procedure described above. In this case no electrode was placed on the separator. It can be seen very clearly, that lamination pressure and temperature cause a flattening and melting of the polymer beads as well as some of the fibres. Especially Fig 2B) shows a good connection between the beads and the separator surface, since the material is pressed into the voids of the rough surface of the separator. This is most desirable for lamination application. After the lamination of the separator to the electrode the separator is pulled off again to investigate the laminated interface.

**Fig 3:**
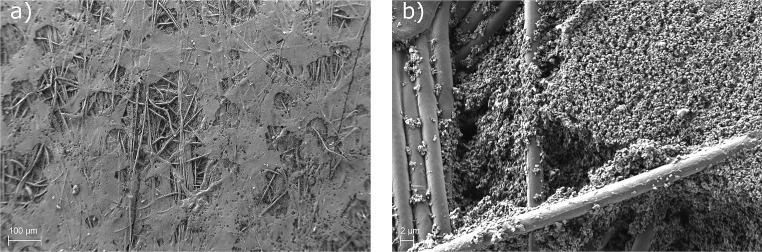
Separator surface after delamination. a) SEM picture of the separator surface after lamination and mechanically induced delamination (100-fold magnification). Craters arising from the delamination can be seen. The shape of the craters resemble the molten and flattened beads in Fig 2. b) Breakage edge at the separator (2000-fold magnification).

**Fig 4:**
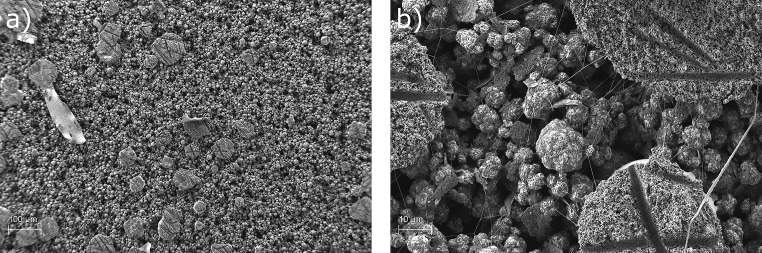
Electrode surface after delamination. a) 100-fold and b) 2000-fold magnification.

The corresponding surface of delaminated separator and electrode are shown in Figs 3 and 4. The separator surface shows great damage after the separation from the electrode. The fibres visible in Fig 3 are part of the initial structure of the separator. Circular spots are visible under SEM, which correlate with the flattened and molten beads seen in Fig 2; these correspond in size and shape. Areas with larger craters originate from agglomerated beads on the separator. Fig 2A) also gives a rough understanding, how much of the separator is blocked by the polymer. This will be important for the later application in battery cell production, since it can impact the electrochemical performance. This will be in the scope of a future paper focusing the electrochemical impacts of this interesting new method. Fig 3B) shows a close-up to the edge the crater. It can be seen that the material of the separator, in this case ceramic particles and binder polymer, is ripped off. This proves a cohesion breakage on the separator. Interestingly, the fibres of the separator are nearly clean from the ceramic/binder system. Polymer from the electrospun coating cannot be found on the separator after delamination. Therefore, samples from the corresponding electrode are also investigated using SEM and shown in Fig 4. Fig 4A) shows the electrode surface after delamination. It can be seen that the surface of the electrode is much rougher than the surface of the separator. The circular spots seen on the surface is separator material transferred to the electrode; as a matter of fact the negative imprints of the fibres inside the separator are visible. Therefore, it can be concluded that the lamination in fact was successful, the critical force is the cohesion force inside the separator material. Interestingly, the fibres connecting the beads after the electrospinning process do not contribute to the lamination and are not visible in Fig 4. The adhesion of the polymer beads to the electrode material is supposed to arise from the melting of the polymer THV. The molten polymer is pressed by the lamination force into the pores of the electrode and afterwards solidifies. To further investigate the lamination effect a manual peeling test using the above described set-up is used. Two samples are prepared. One sample acts as a reference, meaning the non-modified separator is “laminated” the same way as described above to the electrode. The peeling test gives a value of 0.022 N (±0.002 N). The reference sample even tends to lift off from a slight airflow. The modified separator shows a significantly higher peeling force of 0.527 N (±0.005 N) compared to the reference sample. In addition, the sample studied by SEM is also used for a peeling test. It shows a similar value of 0.561 N (±0.005 N). Therefore it can be concluded that lamination is only successful when the separator is modified by Electrospinning in this study. This is in accordance with the findings in [11] and indicates an improvement in the electrochemical performance similar to [11].

## Conclusions

Electrospinning is used to coat a ceramic separator with PVDF fibres and beads to enable lamination onto a NMC111-cathode. Changes of electrode and separator surfaces due to lamination are investigated with SEM imaging. Laminating the coated separator without an electrode shows that the applied temperature and pressure link the polymer beads and the thicker fibres to the surface of the separator in a sufficient way. The phenomenon of cohesion breakage is only seen in case of a laminated separator with an electrospun coating. The conducted peeling test shows that only in this case, a significant force is needed to peel the separator of the electrode and therefore only in this case cohesion breakage can occur. This is underlined by peeling force tests. Interestingly, the lamination depends more on the beads within the coating instead of the electrospun fibres. The process proves therefore its feasibility to enable lamination for separator materials which are intrinsically unable to laminate. More effort in researching this interesting method will be done by our group in the near future on different material systems as well as a thorough investigation of the impact on the electrochemical performance of cells manufactured with this method.

## Supporting information

S1 Raw Images (PDF)
