# Supplementary material for: Lamination of Separators to Electrodes using Electrospinning
Source: PLoS One. 2020 Jan 28;15(1):e0227903. doi: 10.1371/journal.pone.0227903 (PMC6986742; doi:10.1371/journal.pone.0227903)
Supplement: S1 Raw Images — (PDF) [file pone.0227903.s001.pdf]

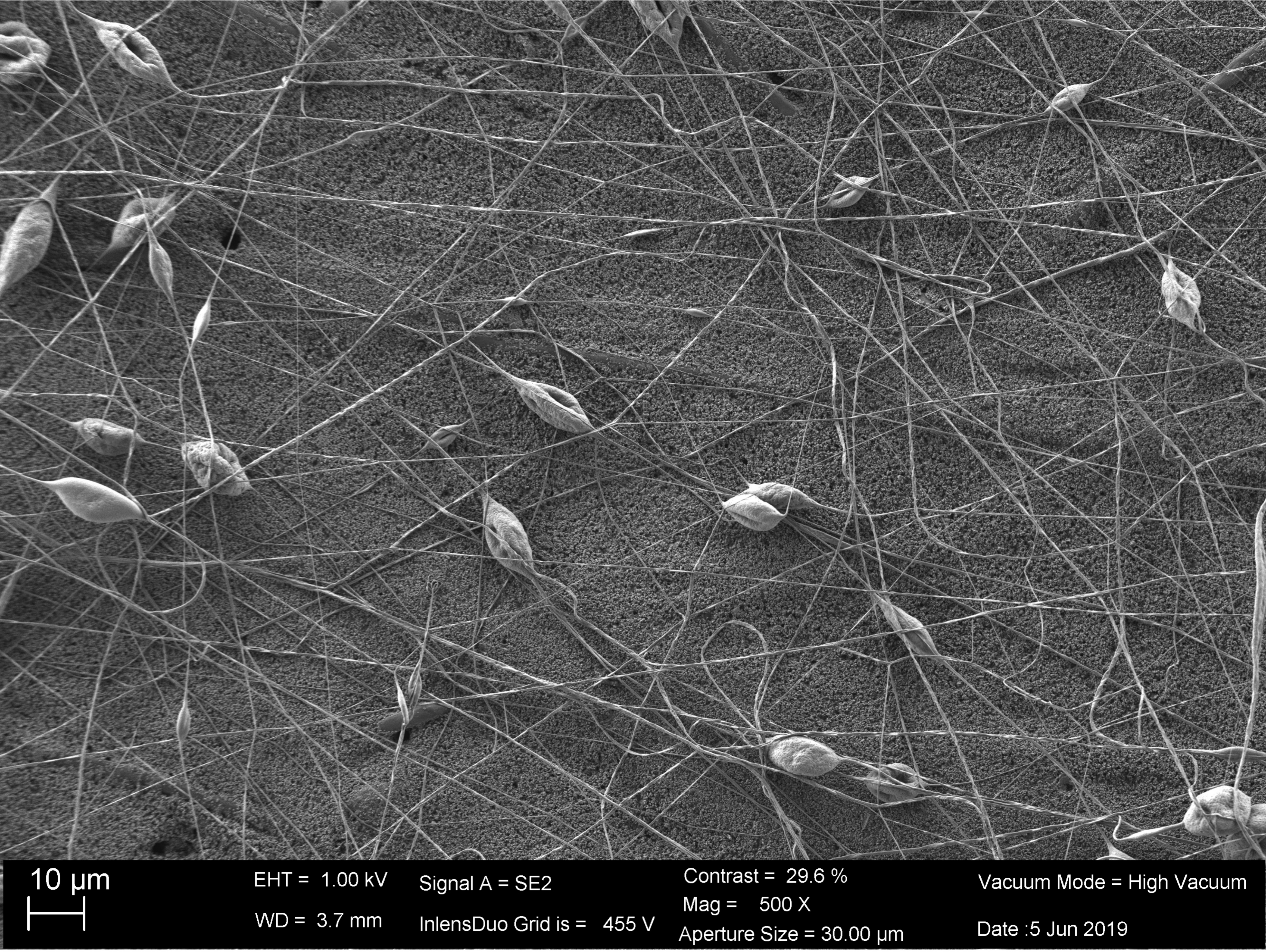

Scanning electron microscopy raw image for figure panel 1 a)

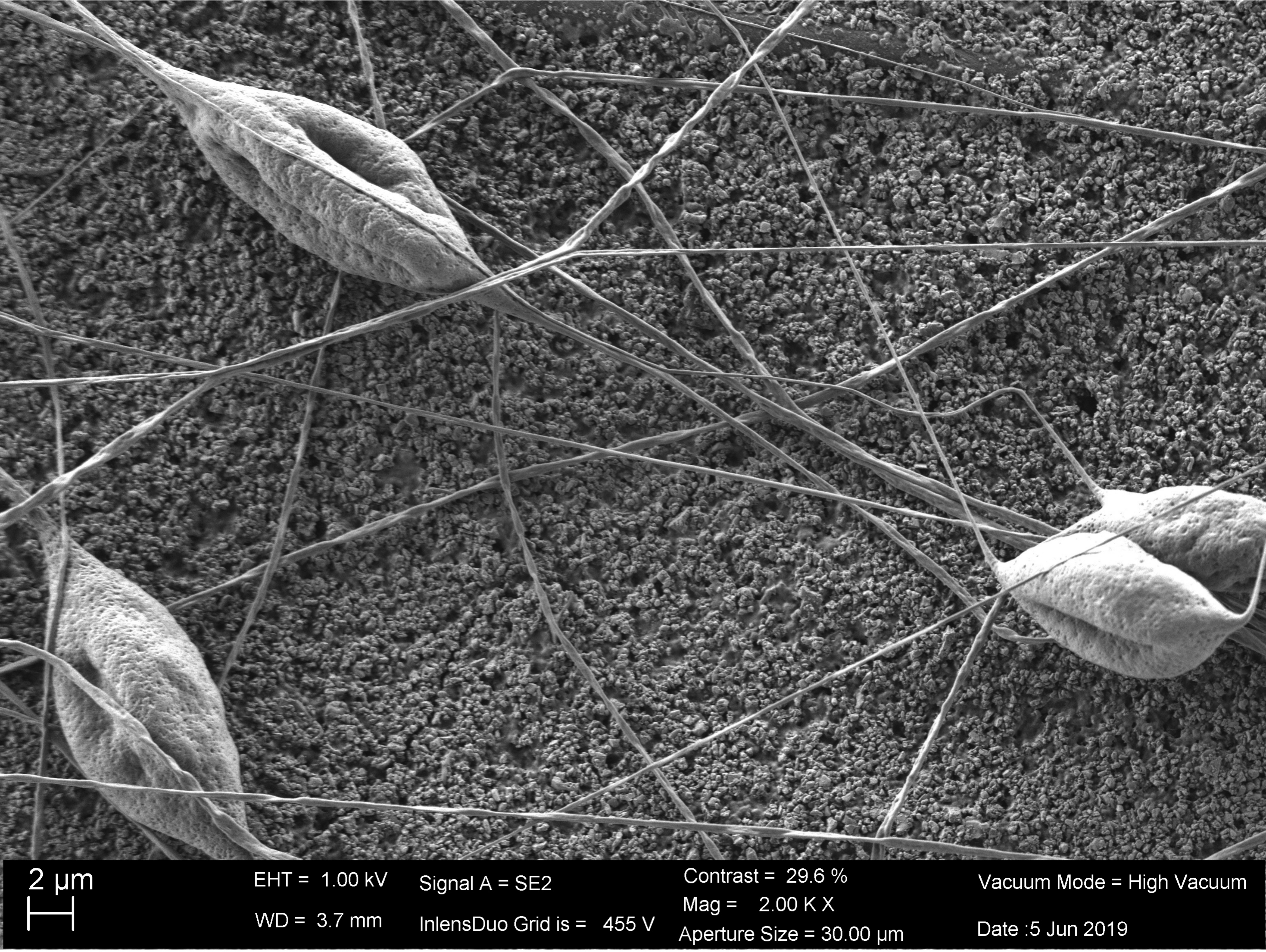

Scanning electron microscopy raw image for figure panel 1 b)

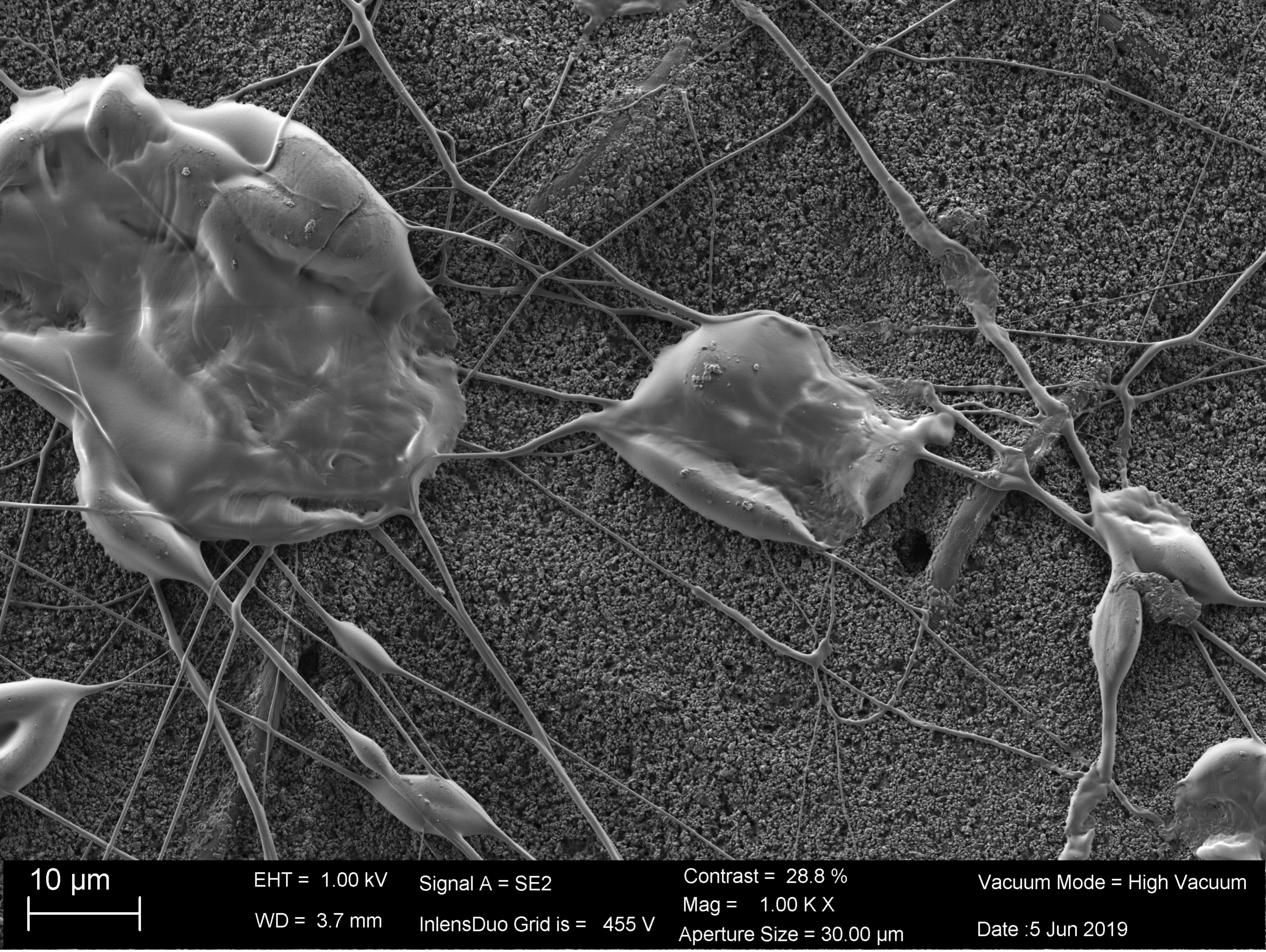

Scanning electron microscopy raw image for figure panel 2 a)

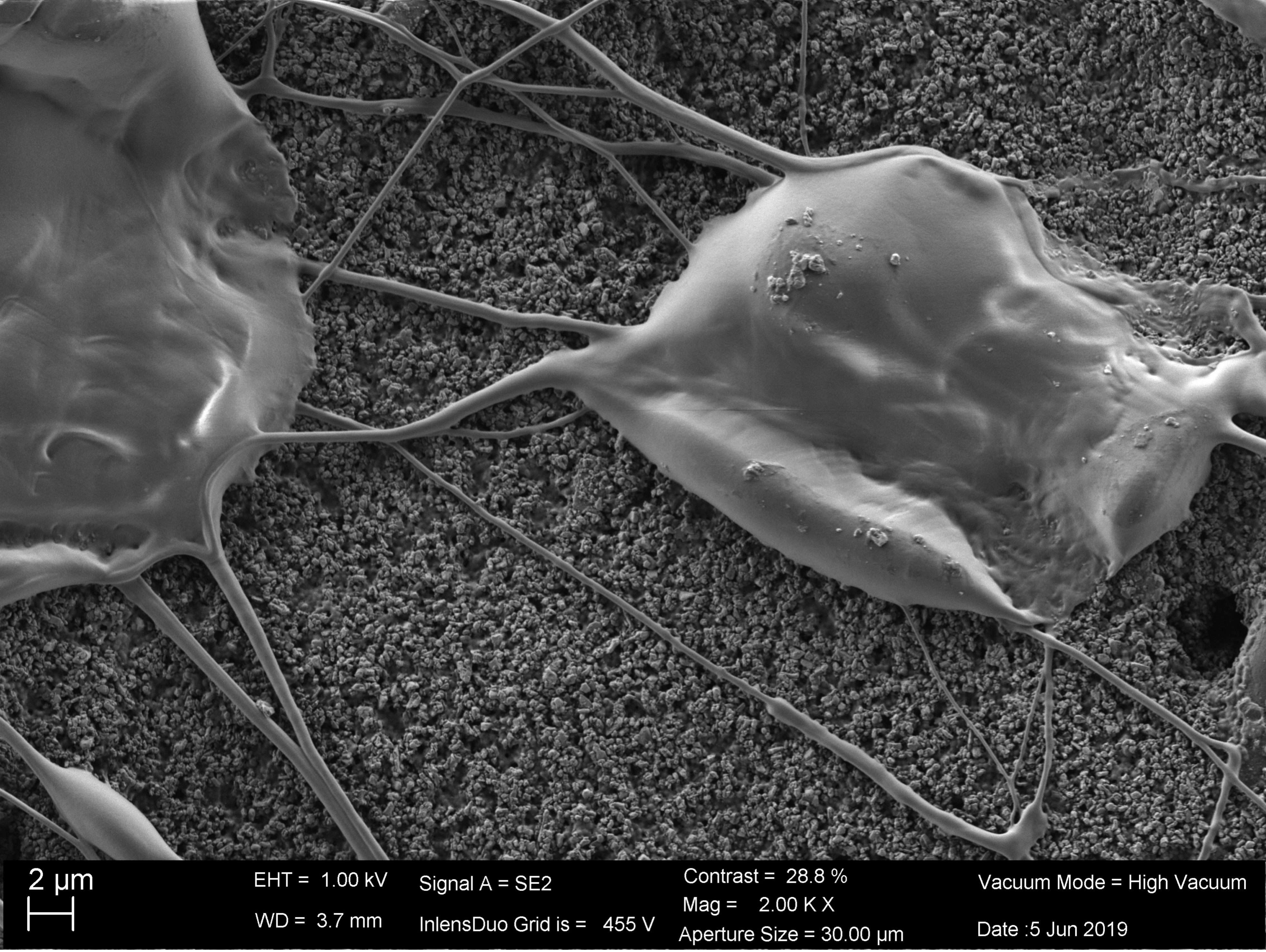

Scanning electron microscopy raw image for figure panel 2 b)

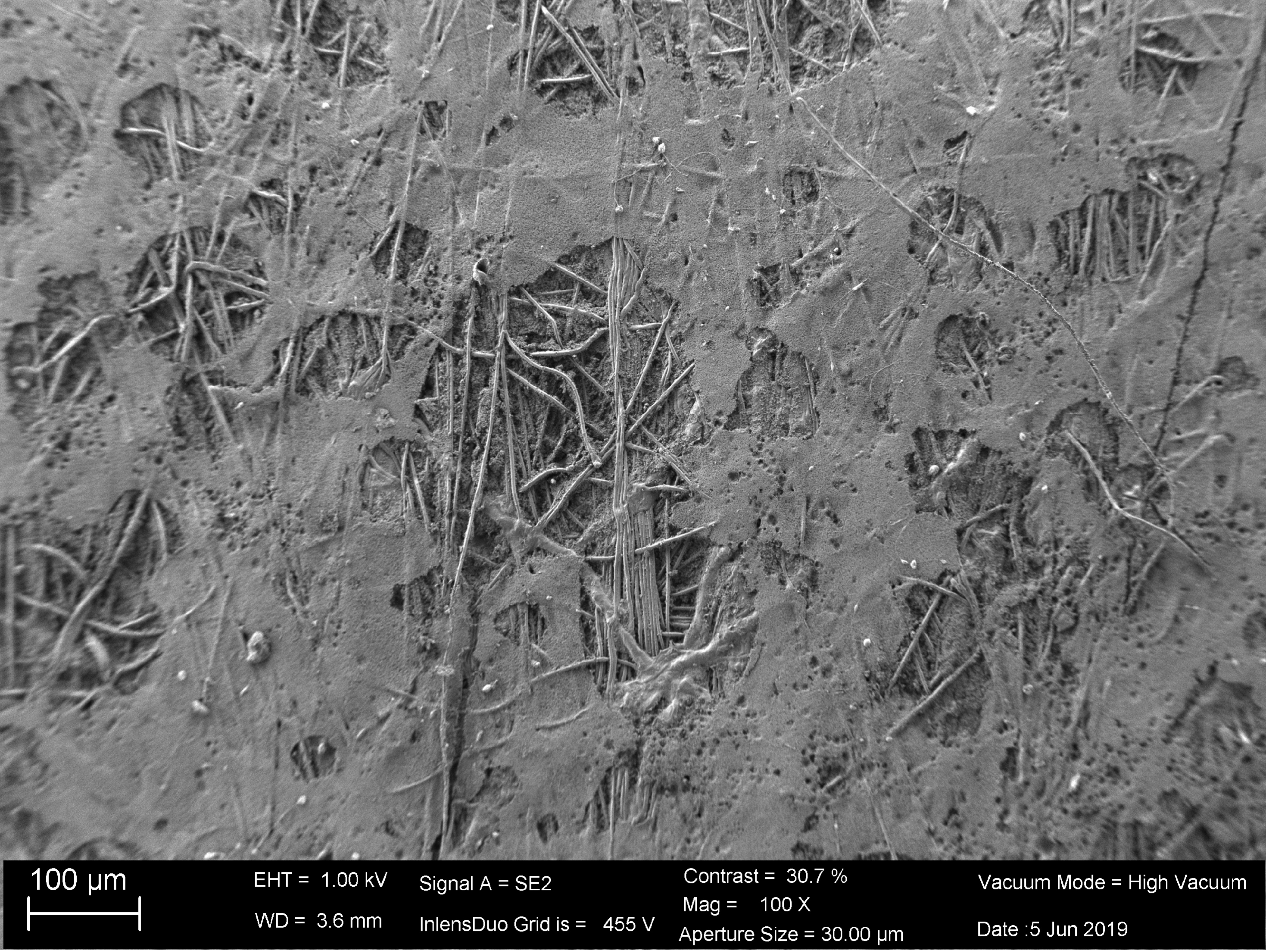

Scanning electron microscopy raw image for figure panel 3 a)

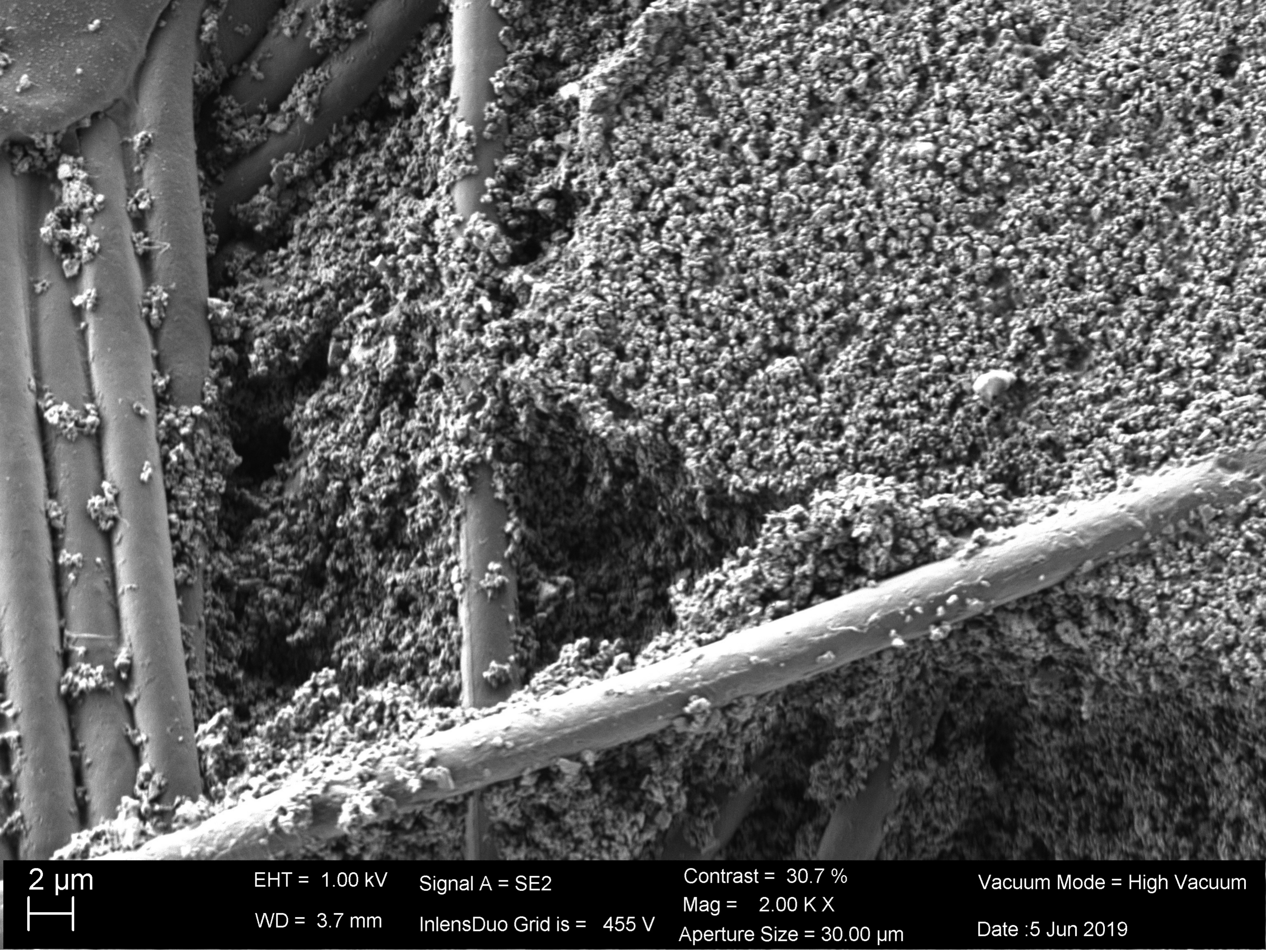

Scanning electron microscopy raw image for figure panel 3 b)

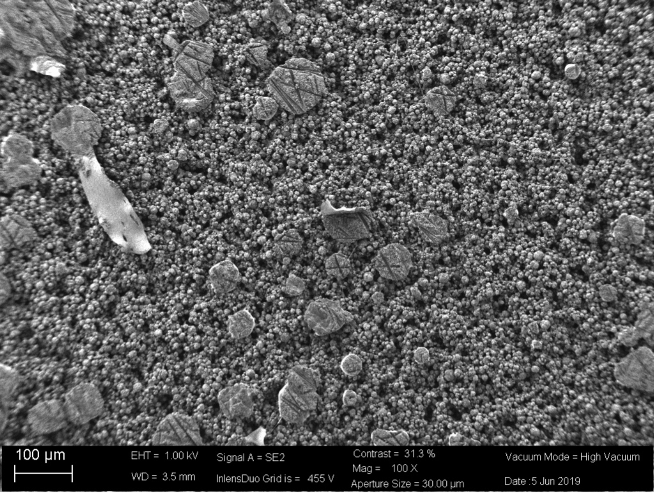

Scanning electron microscopy for figure panel 4 a)

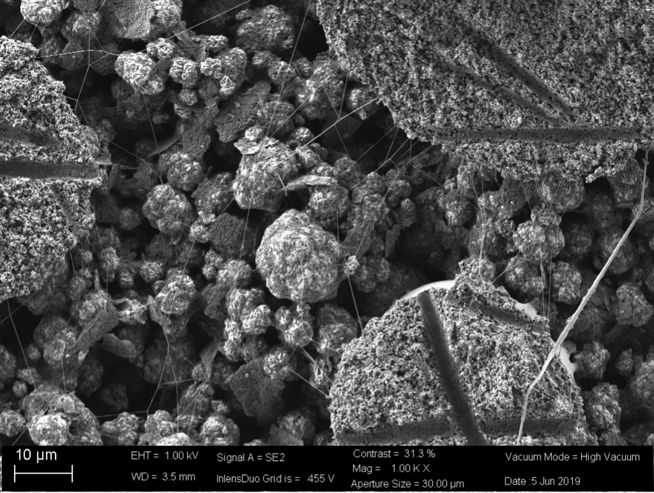

Scanning electron microscopy for figure panel 4 b)
